# Enhanced flight performance in hoverfly migrants

**DOI:** 10.1016/j.isci.2024.111345

**Published:** 2024-11-08

**Authors:** Richard Massy, Will Hawkes, Scarlett Weston, Toby Doyle, Karl R. Wotton

**Affiliations:** 1 Centre for Ecology and Conservation, University of Exeter, Penryn Campus, Cornwall, UK

**Keywords:** ecology, entomology, evolutionary biology

## Abstract

Many animals undergo seasonal migrations in which they travel long distances aided by variations in morphology, physiology, and behavior. Here, we compare the flight characteristics, measured in a tethered flight mill, of autumn migratory and summer non-migratory morphs of the marmalade hoverfly *Episyrphus balteatus* (Diptera: Syrphidae), an ecologically and economically important pollinator, pest predator, and long-distance migrant. Our results show that migratory morphs flew twice as far as the non-migratory morphs. Body condition, reflecting the quantity of energy stores, had an even greater effect as hoverflies with fat abdomens flew almost five times the distance of those with thin abdomens, whereas speed varied only by size. These findings demonstrate enhanced flight capabilities in migratory morphs and underscore the importance of body condition for long-distance flight. Consequently, resource availability, feeding behavior, and the ability to accumulate and utilize fuel are likely to be key factors influencing the migration of hoverflies.

## Highlights

• Migratory hoverflies fly twice as far, but not faster, than non-migratory morphs
• Flight speed only correlated with size, not with migratory status
• Hoverflies with fat abdomens fly almost five times the distance of those with thin abdomens
• Declining resource availability may have major consequences for migratory populations

## Graphical abstract

**Figure:**
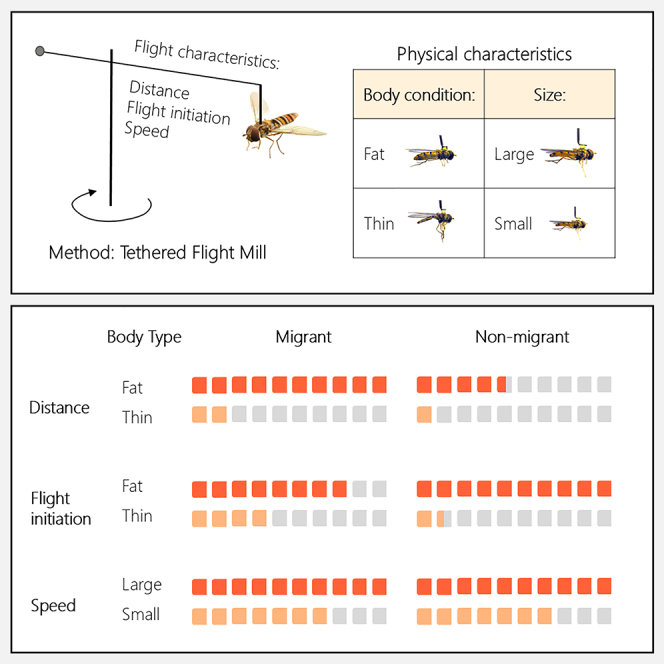

## Introduction

Seasonal migratory behavior combines the temporary suspension of station keeping (routine activities such as foraging and maintenance) with pursuing straighter and longer movement in a seasonally favorable direction.^1^ This change in behavior can result in unusual and risky actions such as movement over large bodies of water including the open ocean,^2^^,^^3^^,^^4^ energetically costly flight against headwinds and within the boundary layer,^5^^,^^6^^,^^7^^,^^8^ or even continuing flight during rainfall,^9^ but offers huge benefits by allowing insects to profit from ephemeral resources,^10^^,^^11^ escape from natural enemies, pathogens,^12^ cold winters, and sweltering summers, and hedge their bets by spreading their offspring over large geographic areas.^13^^,^^14^ Migratory flight in insects is increasingly documented and is likely to be more common than previously thought.^11^^,^^15^^,^^16^

Hoverflies (Diptera: Syrphidae) are a family of flies containing many migratory species that are economically important pollinators and pest predators.^15^^,^^17^^,^^18^ They have been well documented migrating across Europe,^6^^,^^7^^,^^15^^,^^19^^,^^20^ North America,^21^^,^^22^^,^^23^ and increasingly in other regions of the world such as Australia.^24^^,^^25^^,^^26^ Hoverflies are powerful fliers, with a high metabolism capable of supporting sustained flight that is well suited for long-distance migration.^27^ For example, stable isotope studies suggest that the hoverfly *Eupeodes americanus* travels 3,000 km in autumn using a combination of self-powered flight and favorable winds.^22^^,^^28^ The marmalade hoverfly *Episyrphus balteatus* is one of the most abundant migratory hoverflies with a widespread palearctic distribution, with up to three generations per year in Britain.^29^ Like many other migratory organisms, it is a partial migrant since the proportion of migratory individuals varies with latitude,^30^^,^^31^ with the remainder overwintering locally as both larvae and adults.^25^^,^^26^ The autumn generation of adults is longer lived, which in contrast to individuals exposed to summer conditions that typically survive for approximately 1 month under laboratory conditions,^32^ they can last from August to the following March under semi-field conditions.^25^ These mated overwintering females lay the first eggs, which in northern Germany are typically found at the end of March, with a subsequent springtime generation time of ∼45 days.^33^ Although phenology varies by geography, an influx of migrating hoverflies then boost numbers from April in northern China,^34^ through to June in the United Kingdom,^15^ and by July, there is a single panmictic population with no net direction of movement.^15^^,^^26^ *E. balteatus* have been repeatedly recorded heading south from late August^15^ and throughout autumn in the Alps and Pyrenees migrating toward warmer winter habitats,^8^^,^^20^^,^^35^ and making long sea crossings during the multi-generational northward migration in the spring.^2^^,^^3^ Investigating its migration is important to shed light on the seasonal provision of ecosystem services from these hugely abundant pollinators that have increasingly become an important model for the study of animal migration.^28^^,^^36^^,^^37^^,^^38^^,^^39^

Migratory insects often have specialized migratory morphs with enhanced flight capabilities,^40^ increased flight muscle mass,^41^ and a metabolism optimized for efficiency over long distances.^42^ Although exceptions exist, there is a general trade-off between flight proficiency and fecundity even in wing monomorphic species.^43^^,^^44^^,^^45^ As such, migrating insects are often in reproductive diapause where the fecundity is delayed, allowing more energy to be invested in long-distance flight.^46^^,^^47^ Reproductive diapause is elicited by environmental factors such as photoperiod change, temperature, and the quality of food resources^25^^,^^48^^,^^49^ and often occurs in tandem with increased stores of energy by hypertrophy of the fat body.^25^^,^^46^^,^^50^^,^^51^ Fat hypertrophy fuels migration and is common with migrating birds^52^ where body condition is an important factor in determining the behavior, timing, and success of migrations.^53^^,^^54^

The extent to which migratory morphs of hoverflies differ from non-migratory morphs is an area of active study. Transcriptomic analysis of actively migrating hoverflies has demonstrated extensive activation of pathways necessary for the high activity levels associated with long-distance flight,^55^ whereas a comparison of migrant and overwintering morphs showed no difference when flown in a flight mill.^39^ In contrast, the offspring of summer hoverflies showed higher activity (quantified by the time spent walking and flying in an indoor arena) than the offspring of migrating or overwintering morphs,^37^ although the authors of these studies suggested migrants may enter an energy saving state when kept under laboratory conditions, perhaps due to a lack of natural migratory cues. To investigate this discrepancy, we compare the flight characteristics of hoverflies caught at sea level in July, when there is no observed migratory behavior,^5^ with migrating hoverflies caught at high altitude where only migrating individuals are present. We assess how flight performance varies according to size, body-condition morphometrics, and seasonal morph. Despite previous findings, we expect that migratory morphs will fly considerably further than non-migratory summer morphs and that flight distance will correlate positively with body condition, a measure of fuel reserves.

## Results

### Morphometrics

**Figure 1:**
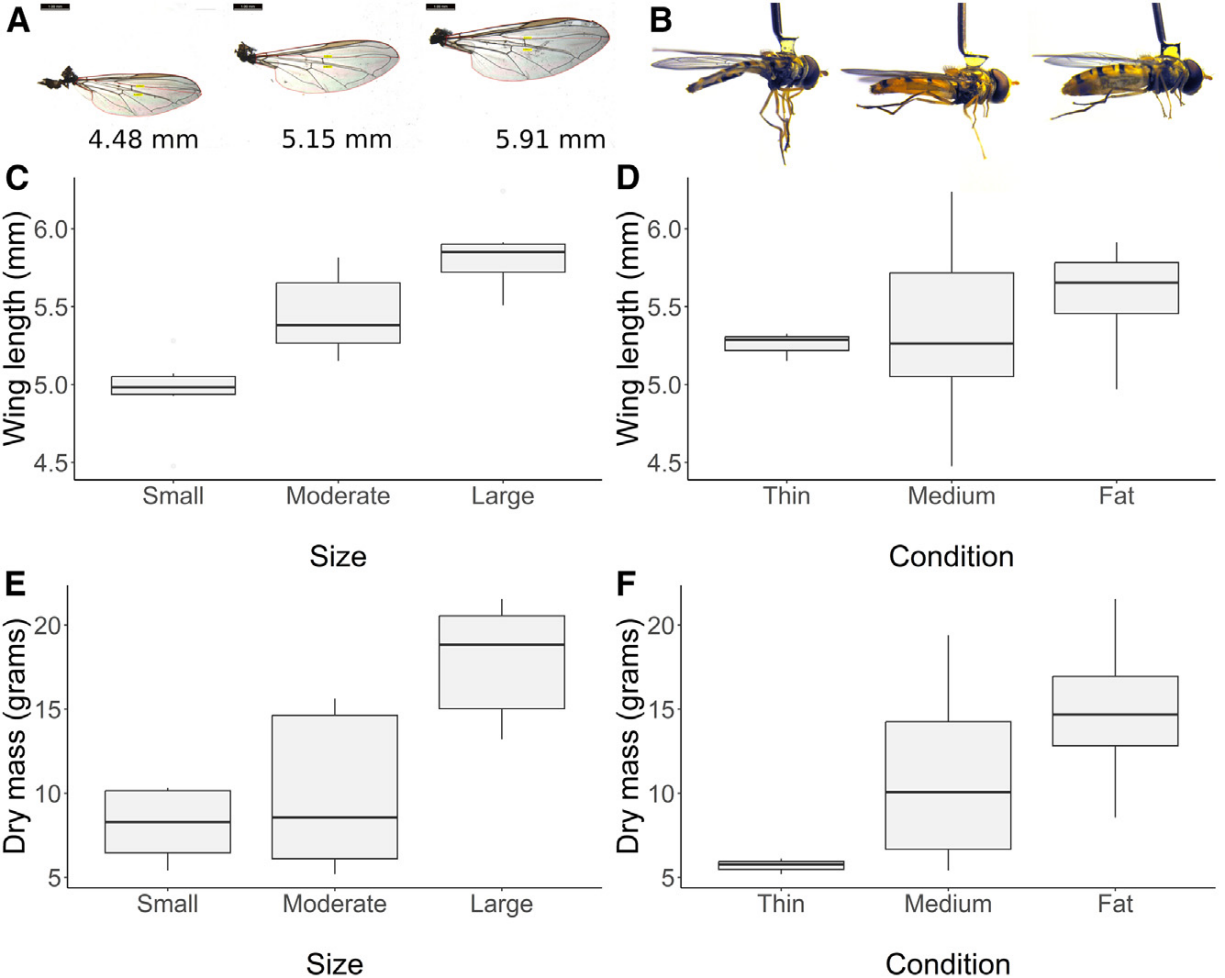
Wing length and weight of migrating *Episyrphus balteatus* hoverflies Size and condition are ordinal measures of insect length and condition (as measured by abdominal plumpness) used to rate hoverflies in the field. (A) Example wings of different body sizes, from left to right: Small, Moderate, Large. 1 mm scale bar for reference. (B) Examples of tethered hoverflies of different conditions, from left to right: Thin, Medium, Fat. (C) Measured wing length by estimated size. (D) Measured wing length by estimated condition. (E) Measured dry mass by estimated size. (F) Measured dry mass by estimated condition.

Ordinal measures were assigned during tethering: size as small, moderate, or large (Figure 1A) and body condition as thin, medium, or fat (Figure 1B). To verify the measures, the dry mass and wing length of 23 migrating hoverflies was measured and then estimated using linear regression with the ordinal measures and their interaction as predictors. Wing length strongly positively correlated with size (Figure 1C) but not body condition (Figure 1D), whereas dry mass strongly positively correlated with both size (Figure 1E) and condition (Figure 1F) although there was no interaction (see Table 1 for parameter estimates). The models represented the available data well as they closely fit the aggregated group means (Figure S1).

**Table 1:** Estimated dry weight (mg) and wing length (mm) of *E. balteatus* hoverflies with different body conditions and wing lengths, with standard error and the sample size in brackets

Weight (milligrams)			
Body condition	Size: small	Size: moderate	Size: large
Thin	3.7_±2.2_ (0)	5.7_±1.6_ (3)	12.8_±2.2_ (0)
Medium	7.5_±1.1_ (5)	9.5_±1.3_ (3)	16.5_±1.2_ (4)
Fat	11.5_±1.6_ (1)	13.5_±1.1_ (5)	20.6_±1.4_ (2)

**Wing length (mm)**			

	Size: small	Size: moderate	Size: large
	4.95_±0.10_ (6)	5.45_±0.07_ (11)	5.84_±0.10_ (6)

Parameter values were obtained from linear models, which explained the majority of the variance. Dry weight: (F_(4,18)_ = 16.18, R^2^ adj = 0.734, *p* < 0.001), wing length: (F_(2,20)_ = 19.71, R^2^ adj = 0.630, *p* < 0.001).

### Overall distance

**Figure 2:**
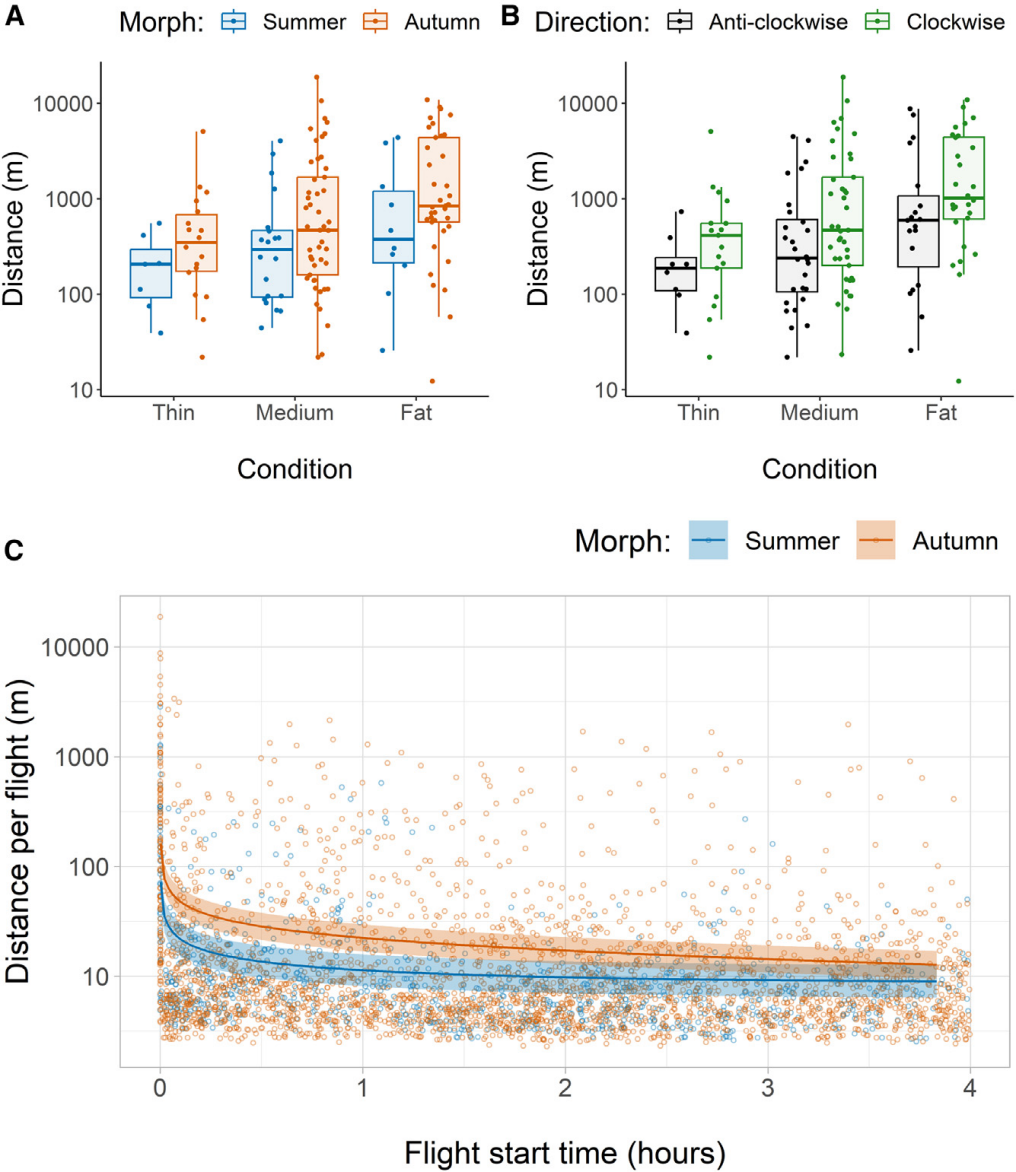
The overall distance flown by hoverflies over 4 h in a flight mill, grouped by: Morph (Summer, Autumn) refers to hoverflies caught during summer or migrating during autumn; Condition (Thin, Medium, Fat) is body condition judged by abdominal plumpness; Direction (Clockwise, Anti-clockwise) denotes the direction of arm rotation (A and B) How distance varies by morph and condition (A) and direction and condition (B). (C) The distance of individual flights over time shown on the log scale with a regression fit (± standard error) weighted by the number of observations of the other variables.

The distance covered by hoverflies over the 4-h experiment varied greatly, ranging from 30 m to 10.6 km (Figures 2A and 2B). Autumn morphs flew approximately twice as far as summer morphs of the same body condition, although condition itself had the greatest impact with fat hoverflies flying 1.6 and 4.6 times as far as medium and thin hoverflies, respectively. Unexpectedly, the direction of rotation also had an effect with clockwise flying hoverflies flying twice as far as those flying anti-clockwise. Size was a poor predictor of distance so was removed from the analysis. The low number of autumn males (*N* = 3) precluded an accurate interaction comparison for sex, but aggregated means showed similar increases in log-transformed distance in the autumn morphs: 112% in males, compared to 131% in females. There was no interaction between condition and direction, so the effect of direction was consistent across all three levels of condition. Despite multiple significant predictors, there remained high residual fly-to-fly variation. Model equation: (Gamma GLM: log(Distance_meters_) = 6.00_±0.29_∗∗∗ + 0.77_±0.29_ x Morph∗∗ + 0.68_±0.26_ x Direction∗ + 1.08_±0.27_ x Condition_linear_∗∗∗ + −0.22_±0.21_ x Condition_quadratic_, N_obs_ = 141_(residual D.F. = 136)_, pseudo R^2^ = 0.186, Gamma dispersion parameter = 2.29). See Table 2 for estimates and Figure S2 for model fits. Summer males had over double the variation of summer females, although an F-test comparing the variances was insignificant: F = 2.15(11,24), *p* = 0.114, CI_0.95_ = 0.830:6.807. See Figure S3 and Table S1 for a female-only comparison.

**Table 2:** The predicted distance, speed, and number of flights over 4 h in a flight mill for 130 hoverflies of different morphs (autumn migratory and summer sedentary), arm-rotation directions, size and body condition (nutrition condition estimated by abdominal plumpness)

Distance covered (meters)				
Direction:	Morph:	Condition: thin	Condition: medium	Condition: fat
Anti-clockwise	Summer	171.8_±68.5_ (3)	482.1_±147.2_ (9)	789.6_±271.1_ (6)
Anti-clockwise	Autumn	371.0_±135.8_ (5)	1041.2_±271.8_ (19)	1705.4_±481.3_ (13)
Clockwise	Summer	339.2_±130.2_ (4)	951.8_±290.6_ (11)	1559_±538..0_ (4)
Clockwise	Autumn	732.6_±236.0_ (13)	2055.8_±458.2_ (30)	3367.1_±841.1_ (24)

**Average speed (meters per second)**				

Direction:		Size: small	Size: moderate	Size: large
Anti-clockwise		0.461_±0.052_ (7)	0.651_±0.045_ (44)	0.674_±0.100_ (4)
Clockwise		0.528_±0.050_ (21)	0.744_±0.046_ (56)	0.771_±0.107_ (9)

**Flight initiation: mean time taken to initiate flight (seconds)**				

Morph:		Condition: thin	Condition: medium	Condition: fat
Summer		3443_±1905_ (7)	1389_±441_ (20)	407_±186_ (10)
Autumn		1175_±394_ (18)	641_±129_ (49)	571_±132_ (37)

Values for distance and speed are back transformed from log model estimates, with the standard error and the respective sample sizes in brackets.

### Distance per flight event

A total of 3,735 separate flights were undertaken by the 141 hoverflies during the 4-h experiments. The difference in flight distance between morphs was greatest in the first 5 s, where the median distance of summer hoverflies was 113 m, compared to 152 for autumn hoverflies. This dropped to 6.86 and 6.82 m, respectively, for flights initiated in the rest of the experiment, although the mean of autumn morphs remained higher than summer morphs due to a smaller number of substantially longer flights (Figure 2C). The data were strongly right skewed, even after log transformation, with a minority of extremely long-distance flights throughout the experiment, but many shorter flights. Neither sex, size, nor the interactions between morph and condition had a noticeable effect on flight distance. Condition had a small but significant positive effect on flight distance, where flights of thin hoverflies were slightly shorter distance, although this had an inverse relationship with time so that by the end of the experiment there was no difference in the distance of individual flights between hoverflies of different conditions. Despite multiple significant predictors, the majority of the variation was due to inter-fly variability (Gamma GLMM: log(Flight distance _meters_) = 1.22_±0.28_∗∗∗ +0.80_±0.25_ x Morph∗∗ +0.89_±0.23_ x Condition_linear_∗∗∗ +0.13_±0.19_ x Condition_quadratic_ + 0.37_±0.30_ x Size_linear_ + −0.25_±0.21_ x Size_quadratic_ + 0.75_±0.22_ x Direction∗∗∗ +0.49_±0.14_ x Time∗∗∗ + −0.31_±0.01_ x TimeLog∗∗∗ + −0.47_±0.13_ x Morph: Time∗∗∗ + −1.02_±0.15_ x Condition_linear_: Time∗∗∗ + −0.28_±0.11_ x Condition_quadratic_: Time∗, N_obs_ = 3607_(residual D.F. = 3593)_, pseudo R^2^_lognormal_ = 0.248 with fixed effects only and 0.789 with random effect of fly_(N=141)_: Time and rotation direction, Gamma dispersion parameter = 0.807).

### Speed

**Figure 3:**
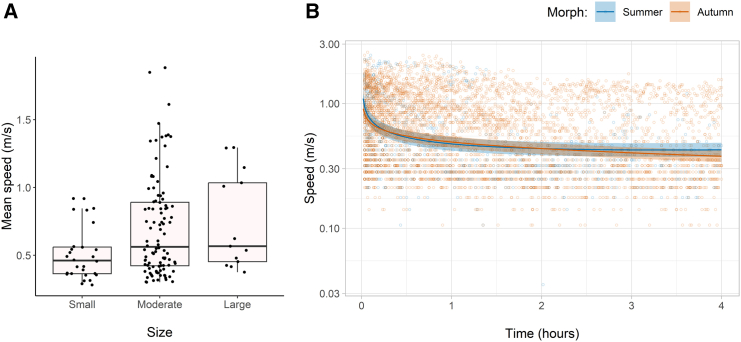
The speed of hoverflies over 4 h in a flight mill (A) How the mean speed varies by estimated size. (B) Hoverfly flight speeds throughout the experimental period, shown on the log scale with regression fit (± standard error) weighted by the number observations of the variables. Speed was deduced from the number photointerrupter events in 1 s, ranging from 1 (0.035 ms^−1^) to 72 (2.54 ms^−1^).

The mean speed of hoverflies over the course of the 4-h experiments varied from 0.28 to 1.88 ms^−1^ but did not significantly differ between summer and autumn morphs, which had medians of 0.62 and 0.51 m per second, respectively. Size was the only significant predictor, with the regression showing that large hoverflies fly 46% faster than small hoverflies and 4% faster than moderate hoverflies, although direction also had a minor effect (Gamma GLM: log(mean speed_meterspersecond_) = −0.53_±0.08_∗∗∗ +0.13_±0.09_ x Direction +0.27_±0.12_ x Size _linear_∗ + −0.13_±0.08_ x Size_quadratic_, N_obs_ = 141_(residual D.F. = 137)_, pseudo R^2^ = 0.087, Gamma dispersion parameter = 0.242) (see Figure 3A).

#### Time transect: Speed

Flight speed was highest at the beginning of the experiment with a median over the first 5 min of 0.84 ms^−1^, which fell linearly to 0.35 ms^−1^ after 2 h before remaining level for the remainder of the experiment (0.39 ms^−1^). Modeling showed that the rate of decrease was similar between autumn and summer hoverflies, although the decrease in autumn hoverflies was more linear than that of summer hoverflies (Figure 3B). Condition had the greatest effect, and interactions with time showed that fat hoverflies begin trials flying at 1.5 times faster than thin and medium hoverflies, although this advantage disappeared from the mid-experiment onward. Size and direction had noticeable effects, with larger and clockwise-flying hoverflies flying slightly faster (Gamma GLM: log(Speed_meterspersecond_) = −1.30_±0.13_∗∗∗ +0.22_±0.12_ x Morph· + 0.32_±0.12_ x Condition_linear_∗∗ + −0.11_±0.09_ x Condition_quadratic_ + 0.19_±0.11_ x Size_linear_· + −0.11_±0.08_ x Size_quadratic_ + 0.15_±0.08_ x Direction· + 0.30_±0.10_ x Time∗∗ + −0.23_±0.02_ x TimeLog∗∗∗ + −0.34_±0.10_ x Morph: Time∗∗∗ +0.07_±0.02_ x Morph: TimeLog∗∗ + −0.34_±0.12_ x Condition_linear_: Time∗∗ +0.00_±0.08_ x Condition_quadratic_: Time +0.01_±0.02_ x Condition _linear_: TimeLog + −0.05_±0.02_ x Condition_quadratic_: TimeLog∗∗, N_obs_ = 5282_(residual D.F. = 5265)_, pseudo R^2^_trigamma_ = 0.171 fixed effects only and 0.692 including random effects of Fly_(N=141)_ and its interaction with time, Gamma dispersion parameter = 0.119).

#### Flight initiation

**Figure 4:**
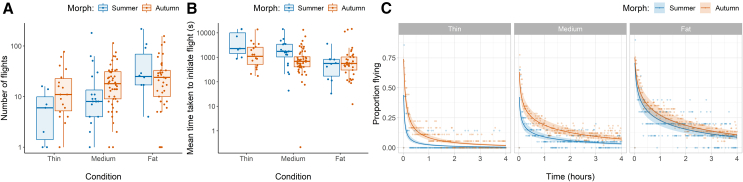
Hoverfly flight activity grouped by morph and body condition (A) The number of flights initiated over the 4-h experimental period. (B) The mean time taken (seconds) for hoverflies to initiate a flight event. (C) The proportion of hoverflies flying at each minute throughout the experimental period, with regression fits (± standard error) weighted by the number of observations of other variables.

Hoverflies undertook between 1 and 216 flights during the 4-h experimental period, with medians of 18.5 and 9.0 flights for autumn and summer hoverflies, respectively (Figure 4A). Autumn hoverflies and hoverflies with fatter abdomens initiated more flights. Due to the additive effect of condition and autumn morphs increasing flight duration, fat autumn hoverflies initiated fewer flights than fat summer hoverflies as long flight durations resulted in fewer individual flight events. Transforming the metric to the time taken to initiate flight showed that there was no difference between fat autumn and summer hoverflies, and an interaction between morph and condition revealed that the effect of condition was about three times greater for summer hoverflies than autumn hoverflies (Linear model: log(Flight initiation_flightsperhour_) = 7.13_±0.25_∗∗∗ + −0.50_±0.29_ x Morph· + −1.51_±0.49_ x Condition_linear_∗∗ + −0.13_±0.38_ x Condition_quadratic_ + 1.00_±0.56_ x Morph: Condition_linear_· + 0.33_±0.44_ x Morph: Condition_quadratic_, Nobs = 141_(residual D.F. = 135)_, R^2^_adj_ = 0.838). See Table 2 for estimates and Figure S3 and Table S1 for a female-only comparison.

#### Time transect: Activity

Flight activity decreased exponentially from 78% at the beginning of the experiment to 8% at the end of the experiment (Figure 4C). Reflecting this rate of decay, the log-transformation of time, referred to as timeLog, proved to be a much better predictor of flight activity than linear time. To avoid overfitting the model, interactions between condition and morph, morph and time, and morph and timeLog were removed, despite lowering model AIC, as their effect sizes were negligible compared to the remaining terms. The resulting regression showed that the probability of flight was higher in autumn morphs and in flies of better condition. Although there was very little difference between fat summer and fat autumn hoverflies, an interaction between morph and condition meant that the effect of condition was higher in summer hoverflies, resulting in vastly reduced activity of medium and especially thin summer hoverflies. The interaction between condition and timeLog reflected a markedly higher flight activity of fat hoverflies at the end of the experiment compared to medium and thin (Binomial GLMM: logit(Proportion flying) = −3.04_±0.29_∗∗∗ +0.79_±0.07_ x Morph∗∗∗ +2.06_±0.14_ x Condition_linear_∗∗∗ + - 0.32_±0.10_ x Condition_quadratic_∗∗∗ + −0.62_±0.12_ x Time∗∗∗ + −0.58_±0.03_ x TimeLog∗∗∗ + −0.75_±0.13_ x Morph: Condition_linear_∗∗∗ + −0.08_±0.09_ x Morph: Condition_quadratic_ + 0.23_±0.04_ x Condition_linear_: TimeLog∗∗∗ + −0.15_±0.03_ x Condition_quadratic_: TimeLog∗∗∗, N_obs_ = 33840_(residual D.F. = 33828)_, pseudo R^2^ (theoretical) = 0.223 for fixed effects only and 0.257 including the random effects of sex and rotation direction).

## Discussion

### Improved flight performance in hoverfly migrants

Long-distance flight is energetically demanding but key to migratory life, facilitating seasonal travel between distant habitats. Here, we demonstrate that *E. balteatus* hoverflies caught during their autumn migration flew over twice as far in flight-mill tests as non-migrants caught during summer. This increased performance is likely to be due to a combination of delayed reproduction in the migrants, which by avoiding the energetic cost of gravidity can increase flight performance and migration-specific traits such as metabolic adaptations controlling the transport and utilization of fuel.^46^^,^^55^^,^^56^ Autumn males experienced a similar increase in flight distance but their low numbers, along with a lack of data from non-gravid summer female morphs, mean that the relative contributions of delayed-reproduction and other migration-specific traits cannot be definitively answered from the data alone.

Previous flight mill experiments with *E. balteatus* by Dällenbach et al., 2018^39^ found no difference in the flight duration (a proxy for distance) or the number of flights initiated between wild-caught autumn migratory hoverflies (caught at the Col de Bretolet, Switzerland in September–October) and overwintering hoverflies (caught around Bern in April) and suggest that migrating hoverflies enter into an energy saving state under laboratory conditions, although it is possible that the spring phenotype is also migratory. If the April-caught hoverflies were under reproductive diapause, equal performance between migratory and non-migratory overwintering hoverflies may suggest that the differences observed in this study were due to the energetic cost of gravidity in the summer population. An assumption of both studies, however, is that the study animals remain in migratory mode during their time in captivity (this study had a maximum duration in captivity of 10 days, with a median of 4 days in the autumn population). Although the flight performance of the autumn population in this study might have been attenuated by loss of migratory behavior, the enhanced flight performance seen suggests that loss of performance, if any, was only partial.

### Behavioral factors affecting flight performance

Flight direction, although recorded to eliminate directional bias and help identify flight events, found that clockwise spinning flies outperform anti-clockwise spinning flies in distance flown and to a lesser extent speed. It is unlikely that tethering differences or asymmetry in wing size (which is widespread among insects,^57^ although their flexible wings reduce steering asymmetry^58^) could have introduced the discrepancy between directions as the hoverfly thrust vectors would have had to be off-positioned by 29° to account for the 14% difference in speed or by 60° to account for the 97% difference in distance. This suggests the difference between clockwise and anti-clockwise directions is behavioral, highlighting that flight performance is a function of both physical ability and motivation to fly. Differing motivation is apparent in the flight activity and flight initiation metrics where autumn migratory morphs had increased flight time and flight initiation. This is likely to be due to migratory restlessness, which is widely reported to increase flight initiation in avian literature^59^ and the activity of *Episyrphus balteatus*.^37^ Although autumn flies with fat abdomens initiated twice as many flights as thin flies, summer morphs were much more affected by condition, with fat flies initiating over eight times more flights than thin flies. The difference between morphs is such that autumn morphs were more motivated to fly with low energy reserves than their summer counterparts. The lack of local food resources at their place of capture might necessitate continued migratory flight to reach suitable stopover habitat for foraging, whereas low-condition summer morphs were captured in the proximity of food resources so might be more content to wait for more optimal foraging conditions (such as a sunny day or airborne flower scents).

### Migration is a marathon, not a sprint

Hoverfly size was the strongest predictor of flight speed, with small hoverflies being considerably slower than moderate and large hoverflies. It was surprising to find no difference in speed between autumn and summer morphs. In contrast to primary flight behaviors that incorporate searching such as foraging, which are likely to prioritize time aloft, migratory flight should be optimized to minimize the cost of transport and therefore be expected to have a higher speed to maximize energy efficiency.^60^ Monarch butterflies reared under autumn-like conditions also showed no difference in flight speed to those reared under summer conditions.^42^ They did, however, display a higher flight efficiency and better post-flight recovery,^42^ and comparisons of power and efficiency show that migratory monarchs had more efficient flight than monarchs that were reproductively active.^61^ Therefore, it is possible that migrating hoverflies also throttle their speed to an energy-efficient cruising speed, and our results suggest that adaptations are geared toward endurance rather than power (i.e., flying further but not faster). Although a healthy level of flight activity promotes longevity in monarch butterflies^62^ and Drosophila^63^ compared to low or no activity, artificially elevated flight activity was deleterious to longevity in Drosophila, and in honeybees, the accumulative stress of 10 days of energetic foraging flight also reduces lifespan.^64^ Even with efficient navigation by time-compensating for the changing solar azimuth, a hypothetical autumn migration would take 11 days,^65^ so the demands of long-distance migratory flight might be such that physical constraints such as oxidative stress and muscle wear could limit speeds to lower than the energetic optimum. This could be especially true in hoverflies because reproduction is dependent on continued survival until the following spring, which would likely require ending migration in good physical condition.

### Conclusion

Our results and those of Dällenbach et al.^39^ suggest that migrant hoverflies have superior flight capability than do summer morphs, enabling them to undertake remarkable long-distance migrations. Body condition played the greatest role in determining flight capability, that was expected as individuals with higher energy stores would be able to fly for longer, but the interaction with morph shows that migratory hoverflies might have increased motivation and adaptations to capitalize on their limited stores. An explanation for the superior flight capability of migrants is that delayed reproduction allows them to channel resources into increasing flight capability.^46^^,^^56^ Increased flight ability, also found in overwintering hoverfly morphs, could introduce advantageous behavioral plasticity that allows them to respond to changeable autumn weather conditions, allowing them to migrate when the opportunity arises or increase their mobility to profit from sparse food resources when feeding on clear days. The highly mobile life history of hoverflies suggests that the evolution of migratory behavior may be relatively straight forward by combining long-distance movement with a seasonally favorable direction, as even non-migratory hoverflies are powerful fliers that can travel long distances between resource patches.^17^ Underlying mechanisms to achieve migration could be the expression of flight and metabolic modulators, which are differentially regulated in migrants,^55^^,^^66^ and aid their ability^67^ and motivation^68^ to achieve long-distance flight. Taken together, our findings highlight the importance of body condition, and therefore of resource availability, for the success of migratory journeys. Consequently, environmental change, for example in the form of habitat reduction and fragmentation or phenological mismatch, that reduces these resources at critical time points will have major consequences for migratory populations and may be the major driver of declining numbers of migratory hoverflies seen in Europe.^69^

### Limitations of the study

The low number of autumn males precluded accurate quantification of the effect and interaction of sex on the flight performance of migrants. Although the male:female ratio of caught migrants was 9:1 in the years 2018–2021, subsequent years had a higher ratio of males raising the possibility of a more accurate sex comparison in future. This study also struggled to discern between physiological and behavioral influences on flight performance, so future studies that combine flight activity with physiological and genetic markers could further discern the origin of the migratory syndrome.

## Resource availability

### Lead contact

Requests for information and resources should be directed to the lead contact, Dr Karl Wotton (k.r.wotton@exeter.ac.uk).

### Materials availability

This study did not generate new materials.

### Data and code availability

• All raw and processed flight mill data can be accessed on Mendeley Data: https://doi.org/10.17632/thxf72xp3n.1.
• All original code is available on Mendeley Data: https://doi.org/10.17632/thxf72xp3n.1, see key resources table, and kept updated on github: https://github.com/RichardMassy/FlightMill/.
• Any additional information required to recreate and/or reanalyze this study is available from the lead contact Dr Karl R. Wotton (k.r.wotton@exeter.ac.uk) upon request.

## Acknowledgments

We would like to thank Jason Chapman for useful feedback during lab meetings, Rochelle Meah and Sian Vincent for their help with the logistics of fieldwork and Ellie Rhodes-Williams for feedback and help with formatting.

This work was supported through grants to K.R.W. from the Royal Society University Research Fellowship scheme (UF150126, URF∖R∖211003). R.M. was supported by the NERC GW4+ Doctoral Training Partnership. T.D. and W.H. were supported by awards to K.R.W. from the Royal Society: a Fellows Enhancement Award (RGF∖EA∖180083) and a Research Grant for Research Fellows (RGF∖R1∖180047), respectively.

## Author contributions

Conceptualization, methodology, and validation: R.M. and K.R.W.; software, formal analysis, investigation, and writing—original draft: R.M.; writing—review & editing: R.M., K.R.W., W.H., S.W., and T.D.; resources: R.M., W.H., S.W., and T.D.; supervision, project administration, and funding acquisition: K.R.W.

## Declaration of interests

The authors declare no competing interests.

## STAR★Methods

### Key resources table

**Table:** 

REAGENT or RESOURCE	SOURCE	IDENTIFIER
**Deposited data**		

Experimental data	This paper	Mendeley Data: https://doi.org/10.17632/thxf72xp3n.1

**Experimental models: Organisms/strains**		

Marmalade hoverflies (*Episyrphus balteatus*)	Wild caught, captured on Penryn campus, Cornwall, UK (summer sedentary morphs) and at the Puerto de Bujaruelo, Pyrenees (autumn migratory morphs).	Taxonomy ID: 286459

**Software and algorithms**		

R v.4.2.1	(R Core Team, 2022)	
R Studio IDE v.2023.09.1	(Posit team, 2022)	
R package: DHARMa v.0.4.6	(Hartig, 2022)	
R package: ggplot2 v.3.4.3	(Wickham, 2016)	
R package: MuMIn v.1.47.1	(Barton, 2022)	
R package: Ckmeans.1d.dp v.4.3.4	(Wang and Song, 2011)	
R package: sigclust v.1.1.0.1	(Huang and Marron, 2022)	
R package: cluster v.2.1.3	(Maechler et al., 2022)	
R package: glmmTMB v.1.1.7	(Brooks, Mollie et al., 2017)	
R package: MASS v.7.3.60	(Venables and Ripley, 2002)	
R scripts for concatenating data from different studies with morphometric data, statistical analysis and graphing, and statistical analysis and graphing of morphometrics	This paper	https://github.com/RichardMassy/FlightMill/; Mendeley Data: https://doi.org/10.17632/thxf72xp3n.1
Python v.3.9.7	Python Software Foundation	https://www.python.org/
Anaconda Software Distribution v.4.12.0		https://docs.anaconda.com/
Spyder IDE v.5.1.5	(Pierre Raybaut, 2009)	Spyder-IDE.org
Python library: pandas v.1.3.4	(McKinney et al., 2010)	https://pandas.pydata.org/
Python library: numpy v.1.21.2	(Harris et al., 2020)	https://numpy.org/
Python library: scipy v.1.9.3	(Virtanen et al., 2020)	https://scipy.org/
Python code for running the flight mills and processing the raw data	This paper	https://github.com/RichardMassy/FlightMill/; Mendeley Data: https://doi.org/10.17632/thxf72xp3n.1

**Other**		

Dual-channel photointerruptor (TCUT1600X01 Vishay®), premounted on PCB (Opto Encoder Click, MikroElektronika).	MikroElektronica	https://uk.rs-online.com/web/p/communication-wireless-development-tools/1745640?gb=s
Coding wheel CAD .dxf files	This paper	Supplementary materials: Data S1

### Experimental model and study participant details

#### Hoverfly collection and husbandry

A total of 93 (3 males, 90 females of which 0 were gravid) migrating *Episyrphus balteatus* hoverflies were caught in September and October of 2021 by sweep netting as they broached the Pyrenean Port de Boucharo mountain pass on the border of France and Spain (42.70388N, -0.06415W). A total of 37 (12 males, 25 females of which 22 were gravid) non-migrating summer hoverflies were caught feeding around Penryn campus in the United Kingdom in July 2022 (50.16969N, -5.12357W). Flies were stored in 30 cm^3^ mesh cages for up to 10 days before experimentation with pollen, a cotton pad with 30% honey – water solution and a cotton pad with 100% water supplied. Cages were stored on a bench next to large south-facing windows which received a high degree of natural light (local photoperiod ∼12 hours sunlight per day). To ensure longevity of the hoverflies by reducing their tendency for flight, cages were covered with white felt during daytime to diffuse the direct sunlight and simulate overcast conditions. At night, cages were covered with black felt to eliminate artificial light.

#### Tethering

Immediately prior to experimentation, flies were placed on a sponge and gently restrained by a weighted plastic mesh. A custom-made pin was then glued to the thorax using ultraviolet curing cement (Bondic™). Custom pins were made from 25 mm lengths of 0.64 mm Ø stainless steel tubing, with one end bent and flattened to form a flat 1 mm lip. Pins were then coupled to the flight mill arm via a 10 mm length of cable sheath and twisted to orientate the fly perpendicular to the arm in either a clockwise or anti-clockwise direction, which was chosen randomly at the beginning of each experiment.

#### Fly morphometrics

After tethering, prior to experimentation, the size and body condition of each fly were judged on a three-point scale. Size was judged to be small, moderate or large and condition was judged by abdominal distension to be thin, medium or fat, see Figure 1B for examples. To verify the ordinal measures, a sample of 23 hoverflies (21 female, 2 male) were collected and stored for 12 months after their experimental trial in the autumn in an Eppendorf® tube with a packet of silica gel to desiccate them. The dry mass of each fly was weighed using a Sartorius QUINTIX35-1S balance, and the length and area of the left wing measured under a bright-field microscope (Leica Camera AG: M165 dissecting microscope with DFC295 camera) using LAS core software, see Figure 1A.

### Method details

#### Flight mill construction

Tethered flight experiments were conducted using a 400 mm squared flight mill arena (Figure S4) containing four rotating arms with radii of 90 mm (Figure S5). Dual-channel photointerruptors permitted recording the direction of arm movement via a laser-cut coding wheel comprised of eight opaque areas separated by transparent spaces of equal size (Figure S5A, Data S1). These were connected to a Raspberry Pi v3b+ which ran a custom Python script to record both opaque to blank and blank to opaque transitions, along with the direction of travel, registering a total of 16 timestamps per rotation.

#### Data collection

Both summer and autumn experiments were conducted indoors between 1pm and 6pm, with summer experiments conducted in an unlit laboratory constant temperature room at 21° and 80% RH, and autumn experiments in a darkened room. To normalise lighting between experiments, a multi-LED light (Radion XR15 G4, EcoTech Marine, run at maximum power 95W) was used for all experiments. It was positioned 140 cm directly above the flight mills facing downwards using a camera tripod mount. A 120 mm length of 150 mm Ø white PVC pipe with a layer of photographic diffuser inside (quarter white, LEE filters) was used to concentrate light onto the experimental setup and blur the individual LEDs.

#### Flight mill data processing

A separate Python script converted the raw data into an analysable format. The time difference between recorded timestamps was used to check for errors: the first pass checked for errors where an erroneous change in direction was recorded, and the second pass utilised “find_peaks()” from the library “scipy” (v 1.9.3) to perform rudimentary signal smoothing to filter out acceleration that was outside of the biological range. In either case the changes were conservative and, if needed, used flanking data to fill in any gaps. Flight parameters such as speed, residual speed (the change in speed from a rolling mean of 16) and acceleration were then computed. The recording was split up into separate flight events using a soft speed cut-off, which eliminated the majority of datapoints where the hoverflies were not actively flying. Movement in the forwards direction was considered a flight if it was above 0.20 ms^-1^, or 0.1 ms^-1^ if the insect was accelerating above 0.1 ms^-1^ (insect acceleration was calculated by adding the no-load deceleration of the arm to the overall acceleration, which accounts for air and arm resistance).

Aggregate statistics were performed on the separate flights to produce a multitude of variables such as the distance covered, start/ end times or the mean acceleration for each flight. Averaging in this way weights the data by the number of timestamps, a measure of distance, so time-dependant variables such as speed or acceleration were separately weighted by time. The values of these aggregations were then summed or averaged over the entire experimental period to obtain overall information for each fly. An R script then concatenated the output files from different experiments and merged the data by ID with hand-recorded information of date, morph, sex, body condition and size.

### Quantification and statistical analysis

#### Spontaneous flight initiation

The number of flights initiated can indicate migratory restlessness, as migratory organisms are more motivated to fly so will initiate more flights. This metric penalises high-performing flies however, as flies that fly for long periods without stopping might initiate fewer individual flight events, despite having a strong drive to fly. A fair metric for flight initiation was made by dividing the idle (or non-flying) time by the number of flights. The new unit for flight initiation is the average time taken (seconds) to initiate flight, see equation below.

$${Mean\;time\;to\;initate\;flight}_{seconds}=\frac{\left({14400-FlightTime}_{seconds}\right)}{N_{flights}}$$

#### Experiment time transect

To analyse how flight performance changed over the experimental period, a time transect was undertaken by sampling the number of photointerruptor events during the last second of every minute. The presence or absence of photointerruptor events indicated if flies were flying, which was used to measure flight activity. The number of photointerruptor events provides the speed and multiplying by 0.035 converts the unit to metres per second. To be consistent with the other analyses, only data which were designated as a “flight” were included in the sample; this excluded arm rocking and movement events too short to be designated as a flight. To eliminate pseudo-replication, mixed models accounted for inter-fly variation.

#### Statistical analysis

The aims of the analysis in this study are to evaluate the relative importance of the variables in explaining flight performance and to create predictive models that compare the flight performance between different groups of hoverflies. For each metric, the goal is to build and report the single most competitive predictive model. The approach to reporting focuses on the effect sizes and error,^70^ and although the significance of variables is tested and reported on an interval basis. Parameter choice is informed by a conservative information theoretic approach that may include variables that strengthen the model but are not significant to the 95% threshold.^71^ These parameters are referred to as having a “noticeable effect”.

All statistical analyses were undertaken using R version 4.2.1^72^ with R Studio 2023.9.1.^73^ Plotting was undertaken using either base graphics or ggplot2.^74^ The general linear models were made using base R, and mixed model analysis containing random terms were made with the glmmTMB package.^75^ All statistical models were verified by plotting their residuals against the predictors, by checking for overdispersion where applicable and by comparing models to null models. The package DHARMa^76^ enabled more detailed dispersion and residuals checks using testDispersion() and simulateResiduals(). Where relevant, link functions were selected by AIC. Theta and dispersion estimations for Gamma and models were obtained automatically using the inbuilt iterative processes. Pseudo R^2^ for generalised linear models was obtained by dividing model deviances by null deviances, or for mixed models by using r.squaredGLMM() from the package MuMIn.^77^

#### Modelling flight characteristics and model choice

Data for distance and speed were strongly positively skewed so were analysed using generalised linear models with a Gamma distribution and a log link, using the generalised linear model from base R. Flight initiation was analysed with a linear model, although data were first log transformed to fit a gaussian distribution. The proportion of flying hoverflies in the time transect of flight activity was analysed using a binomial mixed-model regression with a logit link. For flight and time transect speed data, there were differing numbers of observations between flies, so to avoid pseudo-replication and bias, mixed-model regression fitted using maximum likelihood accounted for fly-to-fly variation by using individual as a random term.

When analysing the distance of individual flights, to reduce the bias of late flights being cropped by the experiment end, flights initiated during the final ten minutes were excluded from the results which reduced the number of flights cropped by the end of the experiment to three (out of 4491).

#### Variables and variable selection

Common variables of morph (autumn or summer), condition (thin, medium or fat), size (small, moderate, large), sex (male and female) and direction (clockwise or anti-clockwise) were investigated in all statistical models. The baseline (intercept) level of morph is summer, with the reported effect of morph showing the estimate for autumn hoverflies, and the baseline level of direction is anti-clockwise with the reported effect showing the estimate for clockwise. The factors of body condition and size, recorded on a three-point scale, were treated as ordinal for the purposes of regression. The factors still used up two degrees of freedom but instead of making separate estimates for all three levels as is the case with non-ranked factors such as sex and direction, a polynomial regression estimated linear and quadratic components, represented with subscripts; for example: “Size _linear_” and “Condition _quadratic_”. The linear estimate models a positive linear relationship, and the quadratic estimate models a positive curve; see Table S2 for the multiplicative relationship with the parameter estimates.

Time was use as a parameter in models investigating changes throughout the experiment, although to aid model fits and interpretation, it was normalised from 0-14400 seconds (the four-hour experiment duration) to 0-1. Time was also log transformed and added as an additional parameter (Timelog) to allow the regression to fit a non-linear curve. Biologically-informed interactions were investigated as additional variables where relevant, which included the interactions between morph: condition, morph: sex, morph: time/ TimeLog, and condition: time/ TimeLog. In each model, a step-wise approach was used to eliminate variables that were found to worsen the model fit, as measured by AIC. A two-step approach was taken with interaction terms with a ΔAIC of less than four to investigate if the model would be better without the interaction term as well as one of its components. The starting model, and sequence of variable selection and ΔAIC of each step are available to view in Table S3.

## Supplemental information

Document S1. Figures S1–S5 and Tables S1–S3

Data S1. CAD design for photointerrupter coding wheel

## References

[1] Dingle H., Drake V.A. (2007). What Is Migration?. Bioscience.

[2] Hawkes W.L.S., Walliker E., Gao B., Forster O., Lacey K., Doyle T., Massy R., Roberts N.W., Reynolds D.R., Özden Ö. (2022). Huge spring migrations of insects from the Middle East to Europe: quantifying the migratory assemblage and ecosystem services. Ecography.

[3] Hawkes W.L., Weston S.T., Cook H., Doyle T., Massy R., Guri E.J., Wotton Jimenez R.E., Wotton K.R. (2022). Migratory hoverflies orientate north during spring migration. Biol. Lett..

[4] Alves R.J.V., Costa L.A.A., Soares A., Silva N.G., Pinto Â.P. (2019). Open ocean nocturnal insect migration in the Brazilian South Atlantic with comments on flight endurance. PeerJ.

[5] Gao B., Wotton K.R., Hawkes W.L.S., Menz M.H.M., Reynolds D.R., Zhai B.-P., Hu G., Chapman J.W. (2020). Adaptive strategies of high-flying migratory hoverflies in response to wind currents. Proc. Biol. Sci..

[6] Massy R., Hawkes W.L.S., Doyle T., Troscianko J., Menz M.H.M., Roberts N.W., Chapman J.W., Wotton K.R. (2021). Hoverflies use a time-compensated sun compass to orientate during autumn migration. Proc. Biol. Sci..

[7] Lack D., Lack E. (1951). Migration of Insects and Birds Through a Pyrenean Pass. J. Anim. Ecol..

[8] Aubert J., Goeldlin J., Lyon P. (1969). Essais de marquage et de reprise d’insectes migrateurs en automne 1968. Mitt. Schweiz. Entomol. Ges..

[9] Wainwright C.E., Volponi S.N., Stepanian P.M., Reynolds D.R., Richter D.H. (2023). Using cloud radar to investigate the effect of rainfall on migratory insect flight. Methods Ecol. Evol..

[10] Chapman J.W., Bell J.R., Burgin L.E., Reynolds D.R., Pettersson L.B., Hill J.K., Bonsall M.B., Thomas J.A. (2012). Seasonal migration to high latitudes results in major reproductive benefits in an insect. Proc. Natl. Acad. Sci. USA.

[11] Satterfield D.A., Sillett T.S., Chapman J.W., Altizer S., Marra P.P. (2020). Seasonal insect migrations: massive, influential, and overlooked. Front. Ecol. Environ..

[12] Satterfield D.A., Maerz J.C., Hunter M.D., Flockhart D.T.T., Hobson K.A., Norris D.R., Streit H., de Roode J.C., Altizer S. (2018). Migratory monarchs that encounter resident monarchs show life-history differences and higher rates of parasite infection. Ecol. Lett..

[13] Holland R.A., Wikelski M., Wilcove D.S. (2006). How and Why Do Insects Migrate?. Science.

[14] García-Berro A., Talla V., Vila R., Wai H.K., Shipilina D., Chan K.G., Pierce N.E., Backström N., Talavera G. (2023). Migratory behaviour is positively associated with genetic diversity in butterflies. Mol. Ecol..

[15] Wotton K.R., Gao B., Menz M.H.M., Morris R.K.A., Ball S.G., Lim K.S., Reynolds D.R., Hu G., Chapman J.W. (2019). Mass Seasonal Migrations of Hoverflies Provide Extensive Pollination and Crop Protection Services. Curr. Biol..

[16] Hu G., Lim K.S., Horvitz N., Clark S.J., Reynolds D.R., Sapir N., Chapman J.W. (2016). Mass seasonal bioflows of high-flying insect migrants. Science.

[17] Doyle T., Hawkes W.L.S., Massy R., Powney G.D., Menz M.H.M., Wotton K.R. (2020). Pollination by hoverflies in the Anthropocene. Proc. Biol. Sci..

[18] Hawkes W.L., Menz M.H., Wotton K.R. (2024). Lords of the flies: Dipteran migrants are diverse, abundant and ecologically important. bioRxiv.

[19] Hlaváček A., Lučan R.K., Hadrava J. (2022). Autumnal migration patterns of hoverflies (Diptera: Syrphidae): interannual variability in timing and sex ratio. PeerJ.

[20] Aubert J., Aubert J.-J., Goeldlin de Tiefenau P. (1976). Douze ans de captures systématiques de Syrphides (Diptères) au col de Bretolet (Alpes valaisannes). Mitt. Schweiz. Entomol. Ges..

[21] Knight S.M., Pitman G.M., Flockhart D.T.T., Norris D.R. (2019). Radio-tracking reveals how wind and temperature influence the pace of daytime insect migration. Biol. Lett..

[22] Clem C.S., Hobson K.A., Harmon-Threatt A.N. (2023). Insights into natal origins of migratory Nearctic hover flies (Diptera: Syrphidae): new evidence from stable isotope (δ 2 H) assignment analyses. Ecography.

[23] Menz M.H.M., Brown B.V., Wotton K.R. (2019). Quantification of migrant hoverfly movements (Diptera: Syrphidae) on the West Coast of North America. R. Soc. Open Sci..

[24] Finch J.T.D., Cook J.M. (2020). Flies on vacation: evidence for the migration of Australian Syrphidae (Diptera). Ecol. Entomol..

[25] Hondelmann P., Poehling H.-M. (2007). Diapause and overwintering of the hoverfly Episyrphus balteatus. Entomol. Exp. Appl..

[26] Raymond L., Plantegenest M., Gauffre B., Sarthou J.-P., Vialatte A. (2013). Lack of Genetic Differentiation between Contrasted Overwintering Strategies of a Major Pest Predator Episyrphus balteatus (Diptera: Syrphidae): Implications for Biocontrol. PLoS One.

[27] Tomlinson S., Menz M.H.M. (2015). Does metabolic rate and evaporative water loss reflect differences in migratory strategy in sexually dimorphic hoverflies?. Comp. Biochem. Physiol. Mol. Integr. Physiol..

[28] Raymond L., Vialatte A., Plantegenest M. (2014). Combination of morphometric and isotopic tools for studying spring migration dynamics in Episyrphus balteatus. Ecosphere.

[29] Hart A.J., Bale J.S., Fenlon J.S. (1997). Developmental threshold, day-degree requirements and voltinism of the aphid predator Episyrphus balteatus (Diptera: Syrphidae). Ann. Appl. Biol..

[30] Menz M.H.M., Reynolds D.R., Gao B., Hu G., Chapman J.W., Wotton K.R. (2019). Mechanisms and Consequences of Partial Migration in Insects. Front. Ecol. Evol..

[31] Chapman B.B., Brönmark C., Nilsson J., Hansson L. (2011). The ecology and evolution of partial migration. Oikos.

[32] Pinheiro L.A., Torres L.M., Raimundo J., Santos S.A.P. (2015). Effects of pollen, sugars and honeydew on lifespan and nutrient levels of Episyrphus balteatus. BioControl.

[33] Tenhumberg B., Poehling H.-M. (1995). Syrphids as natural enemies of cereal aphids in Germany: Aspects of their biology and efficacy in different years and regions. Agric. Ecosyst. Environ..

[34] Jia H., Liu Y., Li X., Li H., Pan Y., Hu C., Zhou X., Wyckhuys K.A.G., Wu K. (2022). Windborne migration amplifies insect-mediated pollination services. Elife.

[35] Hawkes W.L., Doyle T., Massy R., Weston S.T., Davies K., Cornelius E., Collier C., Chapman J.W., Reynolds D.R., Wotton K.R. (2024). The most remarkable migrants—systematic analysis of the Western European insect flyway at a Pyrenean mountain pass. Proc. Biol. Sci..

[36] Hondelmann P. (2007).

[37] Odermatt J., Frommen J.G., Menz M.H. (2017). Consistent behavioural differences between migratory and resident hoverflies. Anim. Behav..

[38] Raymond L., Plantegenest M., Vialatte A. (2013). Migration and dispersal may drive to high genetic variation and significant genetic mixing: the case of two agriculturally important, continental hoverflies ( Episyrphus balteatus and Sphaerophoria scripta ). Mol. Ecol..

[39] Dällenbach L.J., Glauser A., Lim K.S., Chapman J.W., Menz M.H.M. (2018). Higher flight activity in the offspring of migrants compared to residents in a migratory insect. Proc. R. Soc. A B..

[40] Chen H., Wang Y., Huang L., Xu C.-F., Li J.-H., Wang F.-Y., Cheng W., Gao B.-Y., Chapman J.W., Hu G. (2022). Flight Capability and the Low Temperature Threshold of a Chinese Field Population of the Fall Armyworm Spodoptera frugiperda. Insects.

[41] Bhaumik V., Kunte K. (2020). Dispersal and migration have contrasting effects on butterfly flight morphology and reproduction. Biol. Lett..

[42] Schroeder H., Majewska A., Altizer S. (2020). Monarch butterflies reared under autumn-like conditions have more efficient flight and lower post-flight metabolism. Ecol. Entomol..

[43] Tigreros N., Davidowitz G. (2019). Flight-fecundity tradeoffs in wing-monomorphic insects. Adv. In Insect Phys..

[44] Cheng Y., Luo L., Sappington T.W., Jiang X., Zhang L., Frolov A.N. (2016). Onset of Oviposition Triggers Abrupt Reduction in Migratory Flight Behavior and Flight Muscle in the Female Beet Webworm, Loxostege sticticalis. PLoS One.

[45] Ge S., Sun X., He W., Wyckhuys K.A.G., He L., Zhao S., Zhang H., Wu K. (2021). Potential trade-offs between reproduction and migratory flight in Spodoptera frugiperda. J. Insect Physiol..

[46] Rankin M.A., Burchsted J.C.A. (1992). The Cost of Migration in Insects. Annu. Rev. Entomol..

[47] Saunders D.S. (2010).

[48] Goehring L., Oberhauser K.S. (2002). Effects of photoperiod, temperature, and host plant age on induction of reproductive diapause and development time in Danaus plexippus. Ecol. Entomol..

[49] Svensson B.G., Janzon L.-Å. (1984). Why does the hoverfly Metasyrphus corollae migrate?. Ecol. Entomol..

[50] Pener M., Ayali A., Golenser E. (1997). Adipokinetic Hormone and Flight Fuel Related Characteristics of Density-Dependent Locust Phase Polymorphism: A Review. Comp. Biochem. Physiol. Part B: Biochem. Mol. Biol..

[51] James D.G. (1986). Effect of temperature upon energy reserves of the monarch butterfly, Danaus plexippus (L.) (Lepidoptera: Danaidae). Aust. J. Zool..

[52] Ramenofsky M., Wingfield J.C. (2007). Regulation of Migration. Bioscience.

[53] Duijns S., Niles L.J., Dey A., Aubry Y., Friis C., Koch S., Anderson A.M., Smith P.A. (2017). Body condition explains migratory performance of a long-distance migrant. Proc. Biol. Sci..

[54] Anderson A.M., Duijns S., Smith P.A., Friis C., Nol E. (2019). Migration Distance and Body Condition Influence Shorebird Migration Strategies and Stopover Decisions During Southbound Migration. Front. Ecol. Evol..

[55] Doyle T., Jimenez-Guri E., Hawkes W.L.S., Massy R., Mantica F., Permanyer J., Cozzuto L., Hermoso Pulido T., Baril T., Hayward A. (2022). Genome-wide transcriptomic changes reveal the genetic pathways involved in insect migration. Mol. Ecol..

[56] Kent J.W., Rankin M.A. (2001). Heritability and physiological correlates of migratory tendency in the grasshopper Melanoplus sanguinipes. Physiol. Entomol..

[57] Pélabon C., Hansen T.F. (2008). On the adaptive accuracy of directional asymmetry in insect wing size. Evolution.

[58] Meresman Y., Ribak G. (2020). Elastic wing deformations mitigate flapping asymmetry during manoeuvres in rose chafers (Protaetia cuprea). J. Exp. Biol..

[59] Alerstam T., Bäckman J. (2018). Ecology of animal migration. Curr. Biol..

[60] Hill R.W., Wyse G.A., Anderson M. (2012).

[61] Fritzsche McKay A., Ezenwa V.O., Altizer S. (2016). Unravelling the Costs of Flight for Immune Defenses in the Migratory Monarch Butterfly. Integr. Comp. Biol..

[62] Shephard A.M., Hund A.K., Snell-Rood E.C. (2023). Metabolic stress as a driver of life-history plasticity: flight promotes longevity and antioxidant production in monarch butterflies. Proc. Biol. Sci..

[63] Lane S.J., Frankino W.A., Elekonich M.M., Roberts S.P. (2014). The effects of age and lifetime flight behavior on flight capacity in Drosophila melanogaster. J. Exp. Biol..

[64] Margotta J.W., Roberts S.P., Elekonich M.M. (2018). Effects of flight activity and age on oxidative damage in the honey bee, Apis mellifera. J. Exp. Biol..

[65] Massy R., Wotton K.R., Massy R. (2023). The efficiency of varying methods and degrees of time compensation for the solar azimuth. Biol. Lett..

[66] Jones C.M., Papanicolaou A., Mironidis G.K., Vontas J., Yang Y., Lim K.S., Oakeshott J.G., Bass C., Chapman J.W. (2015). Genomewide transcriptional signatures of migratory flight activity in a globally invasive insect pest. Mol. Ecol..

[67] Roeder T. (2020). The control of metabolic traits by octopamine and tyramine in invertebrates. J. Exp. Biol..

[68] Brembs B., Christiansen F., Pflüger H.J., Duch C. (2007). Flight initiation and maintenance deficits in flies with genetically altered biogenic amine levels. J. Neurosci..

[69] Gatter W., Ebenhöh H., Kima R., Gatter W., Scherer F. (2020). 50-jährige Untersuchungen an migrierenden Schwebfliegen, Waffenfliegen und Schlupfwespen belegen extreme Rückgänge (Diptera: Syrphidae, Stratiomyidae; Hymenoptera: Ichneumonidae). Entomol. Zeitschrift.

[70] Popovic G., Mason T.J., Drobniak S.M., Marques T.A., Potts J., Joo R., Altwegg R., Burns C.C.I., McCarthy M.A., Johnston A. (2024). Four principles for improved statistical ecology. Methods Ecol. Evol..

[71] ARNOLD T.W. (2010). Uninformative Parameters and Model Selection Using Akaike’s Information Criterion. J. Wildl. Manage..

[72] R Core Team (2022).

[73] Posit team. RStudio (2022).

[74] Wickham H. (2016).

[75] Brooks M.E., Kristensen K., Benthem K., Magnusson A., Berg C.W., Nielsen A., Skaug H., Mächler M., Bolker B. (2017). glmmTMB Balances Speed and Flexibility Among Packages for Zero-inflated Generalized Linear Mixed Modeling. R J..

[76] Hartig F. (2022). https://cir.nii.ac.jp/crid/1370580229833186830.

[77] Barton K. (2022). https://CRAN.R-project.org/package=MuMIn.

