# Supplementary material for: Enhanced flight performance in hoverfly migrants
Source: iScience. 2024 Nov 8;27(12):111345. doi: 10.1016/j.isci.2024.111345 (PMC11617951; doi:10.1016/j.isci.2024.111345)
Supplement: Document S1. Figures S1–S5 and Tables S1–S3 [file mmc1.pdf]

iScience, Volume *27*

Supplemental information

Enhanced flight performance in hoverfly migrants

Richard Massy, Will Hawkes, Scarlett Weston, Toby Doyle, and Karl R. Wotton

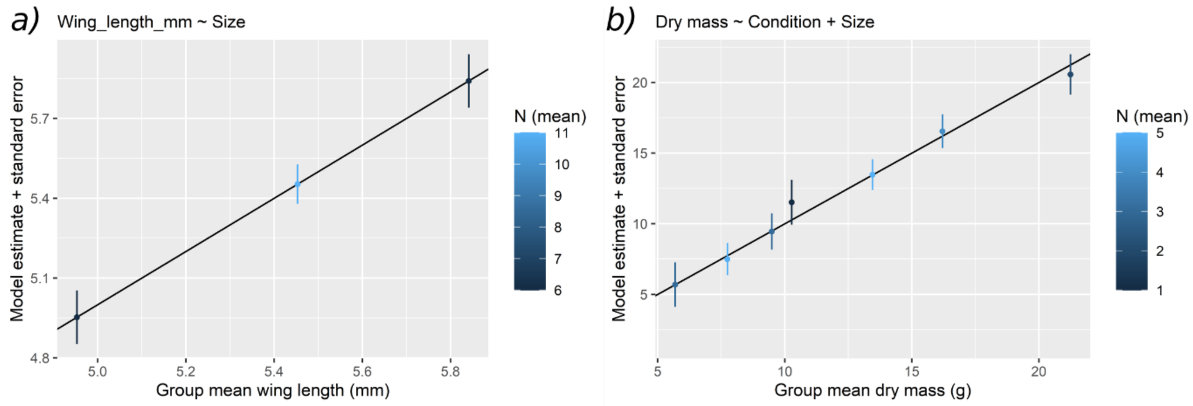

Figure S1: Model fits ± standard error of hoverfly morphometrics plotted against the aggregated means of the factor groups, related to table 1. a) Linear regression of wing length against the assigned characteristic of size (figure 1c in main text). b) Linear regression of dry mass against the assigned characteristics of size and body condition (figure 1e & f in main text).

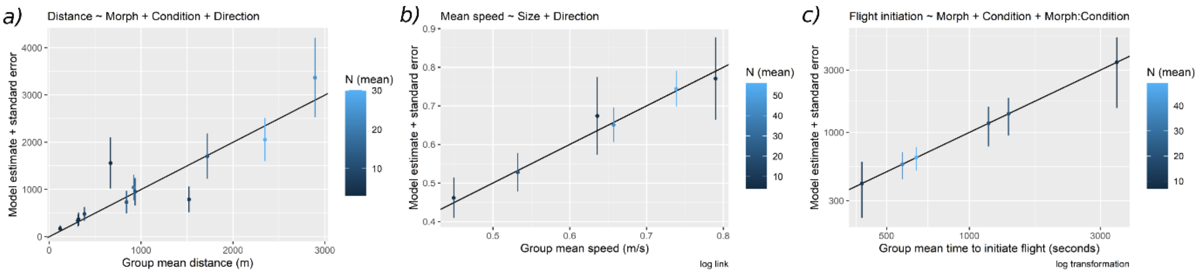

Figure S2: Model fits ± standard error of hoverfly flight parameters, plotted against the aggregated means of the factor groups, related to Table 2. a) A Gamma regression (log link) of the distance travelled (figure 2a, b in main text). b) A Gamma regression (log link) of the mean speed of hoverflies (figure 3a in main text). c) Linear regression (log transformation) of the number of flights initiated per hour of inactivity (figure 4a in main text).

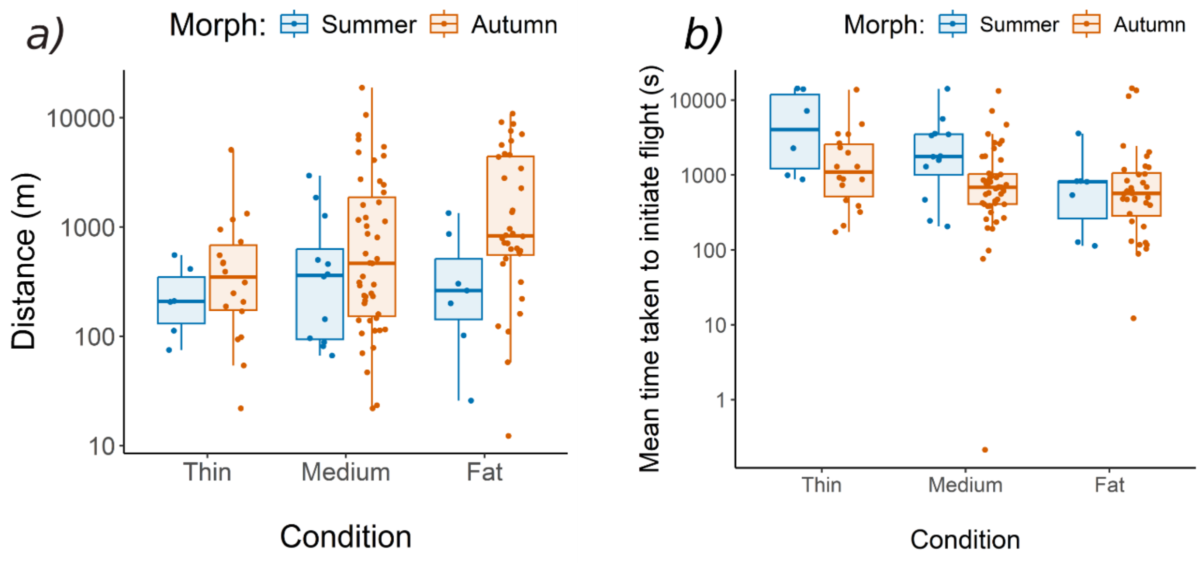

Figure S3: The flight characteristics of female hoverflies, a female-only version of figures 2a and 4a. The distance flown (a) and the mean time (s) to initiate flight (b) of female hoverflies over four hours in a flight mill, grouped by Morph (Summer, Autumn) refers to hoverflies caught during summer or migrating during autumn; Condition (Thin, Medium, Fat) is body condition judged by abdominal plumpness.

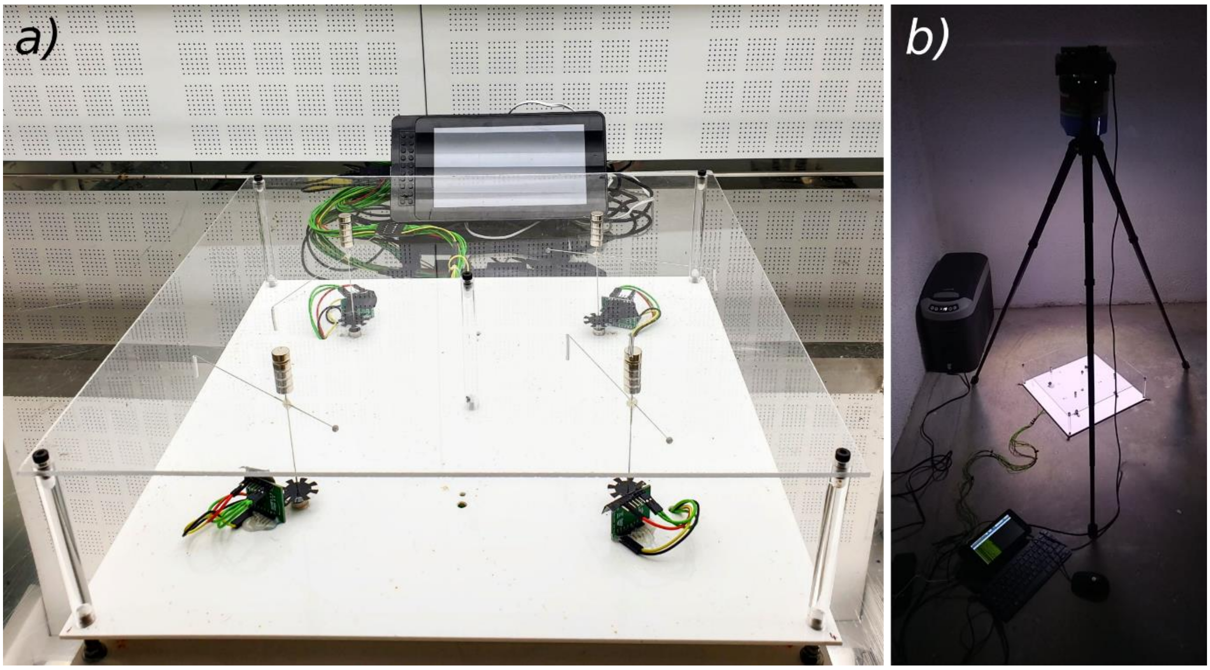

Figure S4: The flight mill apparatus consisting of four flight mills connected to a Raspberry pi computer, related to the STAR methods. The flight mill arena consisted of a 400 mm x 400 mm acrylic sheets, supported by transparent acrylic pillars in which interior threads for M4 bolts were cut, allowing the arena to be assembled and disassembled. Wiring was passed underneath to minimise visual cues. Six 10 mm Ø x 5 mm long ultra-high performance N52 neodymium magnets (Magnet Expert®) were used to support the spindle. The position of the upper magnets could be minutely adjusted to balance the spindle. The plane of polarity was consistent between all magnets in the arena. a) Inspection out of situ. b) Experiment in progress, illuminated by a tripod-mounted aquarium LED lamp.

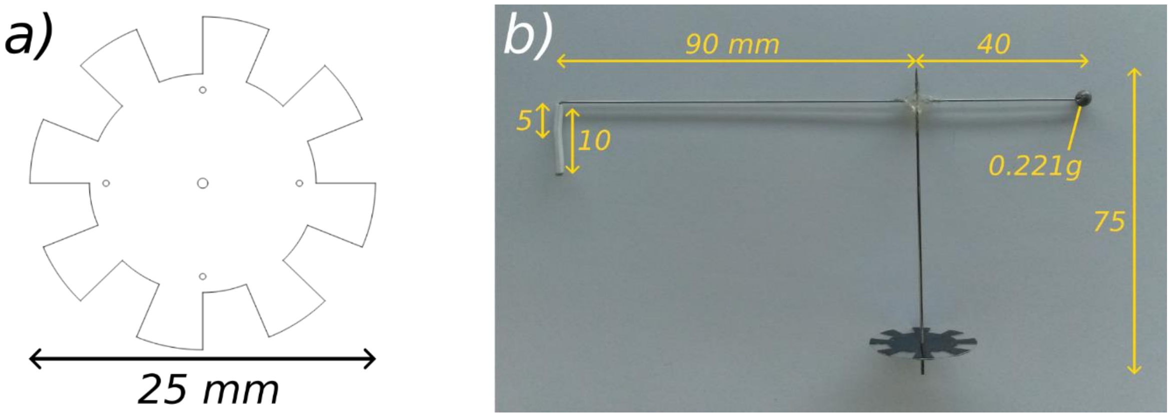

Figure S5: The flight mill arm, related to the STAR methods. a) The CAD design of the photointerruptor coding wheel, which was then laser cut from black card and laminated. b) The photointerruptor spindle with the coding wheel attached. The centre spindles were made from 75 mm steel dressmaker's pins ground down to 74.0 mm and the arms are 135 mm lengths of 0.64 mm Ø stainless steel tubing with a right-angled bend at one end. 10 mm length electronic cable sheath coupled the pinned hoverfly to the arm, which was counterbalanced by a fishing weight attached to the opposite end.

Table S1: The predicted distance and average time taken to initiate flight for female hoverflies, related to Table 2. Values are back transformed from log model estimates, with the standard error and the respective sample sizes in brackets. Distance model (females only): Gamma GLM: log(Distance $_{\text{metres}}$) = $5.64_{\pm0.33}$*** + $1.09_{\pm0.32}$ x Morph** + $0.77_{\pm0.27}$ x Direction** + $0.89_{\pm0.26}$ x Condition $_{\text{linear}}$*** + $-0.34_{\pm0.22}$ x Condition $_{\text{quadratic}}$, Nobs = 126$_{\text{(residual D.F. = 121)}}$, pseudo R^2 = 0.210, Gamma dispersion parameter = 2.09. Flight (females only): Linear model: log(Flight initiation $_{\text{seconds per flight}}$) = $7.32_{\pm0.30}$*** + $-0.70_{\pm0.33}$ x Morph* + -$1.36_{\pm0.56}$ x Condition $_{\text{linear}}$* + $-0.12_{\pm0.46}$ x Condition $_{\text{quadratic}}$ + $0.85_{\pm0.63}$ x Morph: Condition $_{\text{linear}}$ + $0.34_{\pm0.52}$ x Morph: Condition $_{\text{quadratic}}$, Nobs = 126 $_{\text{(residual D.F. = 122)}}$, R^2_{adj} = 0.074.

Distance covered (metres), females only (n=126):				
Direction:	Morph:	Condition: thin	Condition: medium	Condition: fat
Anti-clockwise	Summer	$130.5_{\pm54.6}$ (2)	$370.8_{\pm129.2}$ (6)	$460.4_{\pm174.7}$ (3)
Anti-clockwise	Autumn	$387.7_{\pm140.7}$ (5)	$1101.8_{\pm284.1}$ (18)	$1368.3_{\pm389.2}$ (13)
Clockwise	Summer	$282.8_{\pm111.4}$ (4)	$803.6_{\pm276.3}$ (6)	$997.9_{\pm368.3}$ (4)
Clockwise	Autumn	$840.3_{\pm264}$ (13)	$2388.2_{\pm536.9}$ (29)	$2965.7_{\pm729.3}$ (23)
Flight initiation: mean time to initiate flight (seconds), females only (n = 126):				
Morph:		Condition: thin	Condition: medium	Condition: fat
Summer		$3754.8_{\pm2294.8}$ (6)	$1676.3_{\pm704.8}$ (12)	$551.5_{\pm309.6}$ (7)
Autumn		$1174.5_{\pm399.5}$ (18)	$633.1_{\pm131.8}$ (47)	$575.4_{\pm137.1}$ (36)

Table S2: The ordinal factors of condition and size use a polynomial estimate consisting of a linear and quadratic parts, related to the STAR methods. This table outlines the multiplicative relationship with the parameter estimates, using the parameter of condition as an example.

	Linear	Quadratic
Rank 1 (thin)	- 0.707 * $Condition_{linear}$	0.408 * $Condition_{quadratic}$
Rank 2 (medium)	No change	- 0.816 * $Condition_{quadratic}$
Rank 3 (fat)	0.707 * $Condition_{linear}$	0.408 * $Condition_{quadratic}$

Table S3. Details about the model selection process, related to the STAR methods. The list of variables that were investigated in each starting model, and the relative performance of the removed variables (denoted by the change in AIC).

Starting model	Starting AIC	Drop order and ΔAIC
Distance ~ Morph + Sex + Direction + Condition + Size + Morph: Condition + Morph: Sex	2309.1	Morph: Condition -3.9, Size - 3.6, Morph: Sex -0.1, Sex -1.3
Mean_speed ~ Morph + Sex + Direction + Condition + Size + Morph: Condition + Morph: Sex	58.951	Morph: Condition - 3.7,Condition -2.8, Morph: Sex -1.5, Morph -1.6, Sex -0.47
log(Flight_initiation) ~ Morph + Sex + Direction + Condition + Size + Morph: Condition + Morph: Sex	509.77	Size -2.93, Direction -2.00, Morph: Sex -1.46, Sex -0.95
Flight distance ~ Morph + Condition + Size + Sex + Direction + Time + TimeLog + Morph: Condition + Morph: Sex + Morph: Time + Morph: TimeLog + Condition: Time + Condition: TimeLog	27818.8	Morph: Condition -2.2, Morph: TimeLog -2.0, Morph: Sex -1.8, Sex -1.5, Condition: TimeLog -0.74
Speed ~ Morph + Condition + Size + Sex + Direction + Time + TimeLog + Morph: Condition + Morph: Sex + Morph: Time + Morph: TimeLog + Condition: Time + Condition: TimeLog	-1326.4	Morph: Condition -3.9, Morph: Sex -1.3, Sex -1.6
Flying ~ Morph + Condition + Time + TimeLog + Morph: Condition + Morph: Sex + Morph: Time + Morph: TimeLog + Condition: Time + Condition: TimeLog	25425.8	Condition: Time +28, Morph: Time +3, Morph: TimeLog +1
